# Role of relativity in high-pressure phase transitions of thallium

**DOI:** 10.1038/srep42983

**Published:** 2017-02-20

**Authors:** Komsilp Kotmool, Sudip Chakraborty, Thiti Bovornratanaraks, Rajeev Ahuja

**Affiliations:** 1 Department of Physics, Mahidol Wittayanusorn School, Nakhon Pathom 73170, Thailand; 2 Thailand Center of Excellence in Physics, Commission on Higher Education, Bangkok 10400, Thailand; 3 Condensed Matter Theory Group, Department of Physics and Astronomy, Box 516, Uppsala University, S-75120 Uppsala, Sweden; 4 Extreme Conditions Physics Research Laboratory (ECPRL), Department of Physics, Faculty of Science, Chulalongkorn University, Bangkok 10330, Thailand; 5 Applied Materials Physics, Department of Materials and Engineering, Royal Institute of Technology (KTH), S-100 44 Stockholm, Sweden

## Abstract

We demonstrate the relativistic effects in high-pressure phase transitions of heavy element thallium. The known first phase transition from h.c.p. to f.c.c. is initially investigated by various relativistic levels and exchange-correlation functionals as implemented in FPLO method, as well as scalar relativistic scheme within PAW formalism. The electronic structure calculations are interpreted from the perspective of energetic stability and electronic density of states. The full relativistic scheme (FR) within L(S)DA performs to be the scheme that resembles mostly with experimental results with a transition pressure of 3 GPa. The *s-p* hybridization and the valence-core overlapping of 6*s* and 5*d* states are the primary reasons behind the f.c.c. phase occurrence. A recent proposed phase, i.e., a body-centered tetragonal (b.c.t.) phase, is confirmed with a small distortion from the f.c.c. phase. We have also predicted a reversible b.c.t. → f.c.c. phase transition at 800 GPa. This finding has been suggested that almost all the III-A elements (Ga, In and Tl) exhibit the b.c.t. → f.c.c. phase transition at extremely high pressure.

High-pressure induced phase transition in thallium (Tl) was envisaged over last couple decades^1^,^2^. A structural phase transition of Tl from a hexagonal close packed (h.c.p.) structure to a face-centered cubic (f.c.c.) structure occurs around 4 GPa^2^, and the f.c.c. structure is stable with compressing up to 68 GPa^1^. In recent year, two independent high-pressure studies in Tl over 120 GPa have been reported^3^,^4^. Cazorla *et al*. confirmed the first phase transition from the h.c.p. to f.c.c. structure at 3.5 GPa, and the f.c.c. phase was persistently stable up to 125 GPa based on angle-dispersive x-ray diffraction (ADXRD) method. They also found that the h.c.p. to f.c.c. phase transition was at 8.8 GPa and the f.c.c. phase stabilized up to 4.3 TPa by using the projector augmented wave and generalized gradient approximation (PAW-GGA) method^5^,^6^ with including spin-orbit (SO) coupling based on density functional calculations^3^. In contrast to the Cazorla’s report, Kotmool *et al*. have theoretically studied and predicted the high-pressure phases of Tl up to 200 GPa. The h.c.p. → f.c.c. phase transition has been confirmed at 3 GPa by using L(S)DA with full relativistic calculation based on full-potential with local-orbital minimum-basis set (FPLO) method^7^,^8^. A body-centered tetragonal (b.c.t.) phase has been predicted around 80 GPa, and their experimental results by using the ADXRD method have also insisted the existence of a small distortion of the f.c.c. phase with |(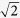 × *a-c*)/*c*| < 1% up to 128 GPa^4^.

The subtle difference of two previous studies^3^,^4^ needs to be clarified in order to have a complete picture of the high-pressure phase transition of the III-A group. As a known b.c.t. → f.c.c. phase transition in gallium (Ga)^9^,^10^ and indium (In)^11^, here an hypothesis of the possible b.c.t. → f.c.c. phase transition beyond 200 GPa in Tl is established. Moreover, as seen in the previous results, a thorough investigation is still needed to make more understanding in high-pressure behavior of this element. Therefore, the present study aims at theoretically investigating the high-pressure phase transitions of Tl up to 1 TPa. The various methods of calculations are employed to find the most appropriate one to represent electronic structure of Tl. An evolutionary algorithm, which is an unbiased method, is used to search the high-pressure phases of Tl beyond 200 GPa up to 1 TPa to proof the initial hypothesis as well.

## Results and Discussion

**Figure 1:**
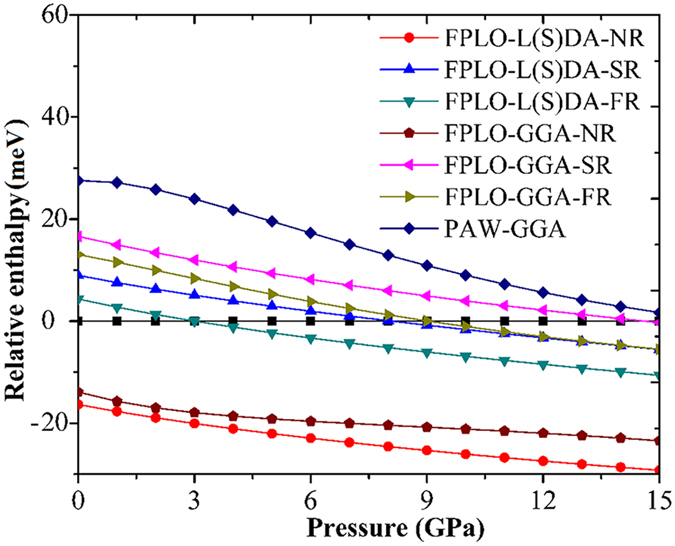
Relationship of relative enthalpies of the f.c.c. phase with grounded by enthalpy of the h.c.p. phase, versus pressure. The calculations are performed using various basis sets, exchange-correlation functionals, and relativistic levels.

Figure 1, shows the relative enthalpy, determined with all considered methods, of the f.c.c. phase from 0 to 15 GPa, with the h.c.p. phase as reference. The results indicate that the relativistic scheme has a strong effect upon the structural phase transition from the h.c.p to f.c.c. But this dependence can not be seen in case of NR calculation within both L(S)DA and GGA schemes. This is because of the fact that the enthalpy of the f.c.c. phase is lower than the h.c.p. phase in that range of pressures. In other cases that consider the SR and FR effects, the h.c.p. → f.c.c. phase transition can be revealed. The result using FPLO with L(S)DA and FR shows reasonable agreement with the experimental outcomes^1^,^2^,^3^,^4^ with a transition pressure of 3 GPa, whereas, the other methods yield the overestimating transition pressures. Interestingly, the transition pressure using GGA and FR within FPLO method is higher than that from the L(S)DA and the PAW-GGA reveals the transition pressure as three times higher than the experimental value. This issue will be discussed later. The fitting parameters and transition pressures of both the phases calculated with other methods are shown in Table 1. For fitting parameters, the results agree following the conventional trend that GGA overestimates the volume and underestimates the bulk modulus, but they are different while using L(S)DA scheme. It is found that the fitting parameters using FPLO with L(S)DA and FR also mostly agree with experiments as well. Therefore, this scheme will be reliable and appropriate option to perform the structural and mechanical properties of thallium.

**Figure 2:**
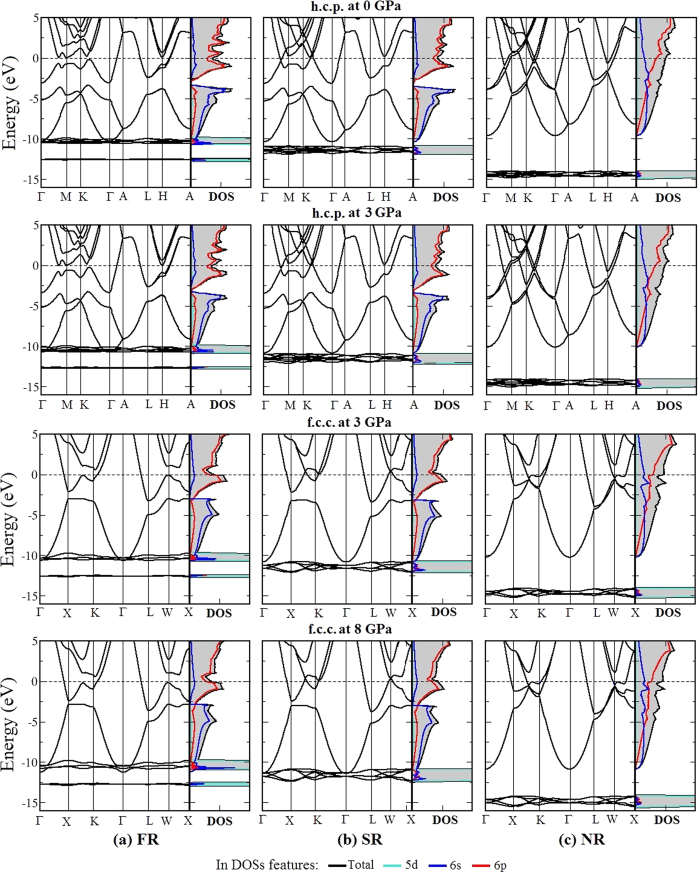
Comparison of electronic band structures and DOSs of h.c.p. phase at 0, and 3 GPa, and f.c.c. at 3 and 8 GPa, by using various relativistic levels, consisting of (column (**a**)) fully relativistic (FR), (column (**b**)) scalar relativistic (SR), and (column (**c**)) non-relativistic (NR) within LS(D)A. The horizontal lines in DOS panels at energy range of −15 to −10 eV, are cut peaks of 5*d* states in which their height are in range of 10–30 state/eV.atom, meanwhile shown scales of DOSs are in range of 0–1 state/eV.atom.

The electronic band structures and density of states (DOSs) of the h.c.p. and f.c.c. phase of Tl at selected pressures as depicted in Fig. 2 show the influence of relativistic effects in particular L(S)DA method. For the h.c.p. phase at 0 GPa (top row), the 6*p* states are the majority across the Fermi level. The DOS shapes for the FR and SR consideration are different in term of smoothness as it is less smooth between 1.0 and 2.0 eV energy range in the former case. This difference also reflects in the energy dispersion of zone-boundary at M- and K-points, where crossing of dispersion lines in the SR is observed, while a small splitting right at H-point in the FR is seen. The DOS and energy dispersions in case of the SR and FR are similar below the Fermi level between −4.0 and −10.0 eV, dominantly occupied by 6*s* states. There is a separation between 6*p* and 6*s* states that is observed around 3 eV below the Fermi level. It leads to the conclusion that there is no *s-p* hybridization, but only bonding of the *p* states exist that forms the h.c.p. phase at ambient condition. In contrast to this separation in case of the SR and FR, both 6*p* and 6*s* states play the important role of bonding electrons in the NR case. This leads to the disagreement with experiment regarding the phase transformation of the h.c.p. → f.c.c. in cases of NR calculations. In the lower energy range between −15 and −10 eV, an apparent difference in relativistic levels is contributed by 5*d* states, which originates together at around −11.8 eV and −14.0 eV in the SR and NR, respectively. But in the FR case, there is a splitting of 5*d* states into 5*d*_5/2_ and 5*d*_3/2_ states as allocated at −10.24 eV and −12.55 eV. This splitting is resulted due to the spin-orbit (SO) coupling in this case, and is in good agreement with the previous experiments^12^,^13^. Furthermore, an overlap of 6*s* and 5*d*_5/2_ at Γ-point is observed in case of FR. The evidences of the h.c.p. → f.c.c. transformation are revealed at 3 GPa, as shown in second and third row of Fig. 2 for h.c.p. and f.c.c., respectively. For the first one, a mixing state between 6*s* and 6*p* appears in the h.c.p. and f.c.c. phase at this pressure for both SR and FR. In contrast, the second evidence of a valence-core overlap of 6*s* and *5d*_5/2_ state appears in the FR case only. The transition pressure for the h.c.p. → f.c.c. phase transition of the FR calculation has been determined as 3 GPa. For the SR calculation, it has been found that the valence-core overlap arises at 3 GPa for the h.c.p. phase and increases with increasing pressure. The bottom row of Fig. 2 displays the band structures and DOS of the f.c.c. phase at 8 GPa (the transition pressure in the SR case) and the overlap of 6*s* and 5*d* states is also appearing in the SR case. In the NR case, the valence-core overlap is unobserved up to 8 GPa as the energy tail of 6*s* states being still far from the 5*d* states.

The calculation results using both FPLO and PAW approach with GGA functional reveal the similar tendency in accord with the previous discussion. But, due to the overestimation of volume and as a consequence to the change in the electronic band structure, the transition pressure is also overestimated using GGA. Moreover, it is found that the use of GGA and FR within FPLO obtains the compatible transition pressure with previous reports using GGA and including SO coupling within PAW method^3^.

The bonding in case of the h.c.p. phase originates from 6*p* states and the valence-core overlapping of 6*s* and 5*d*_5/2_ states is appeared. The external pressure induces *s-p* mixing of valence state and partial-valence state of 5*d* electrons leading to the h.c.p. → f.c.c. phase transformation. This finding corresponds to forming the f.c.c. phase in III-A metal group, consisting of Ga and In as reported in previous investigations^11^,^14^,^15^. It should be emphasized here that the electronic structure of Tl strongly depends on the level of relativistic application.

**Figure 3:**
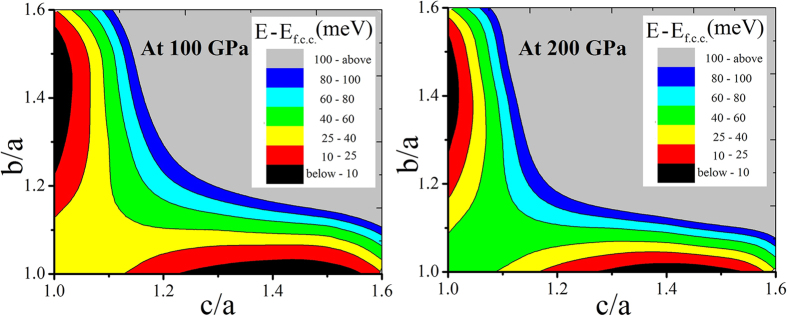
The contour plot of enthalpy of Tl by varying *b/a* and *c/a* at given volumes corresponding to 100 GPa (left panel) and 200 GPa (right panel).

**Figure 4:**
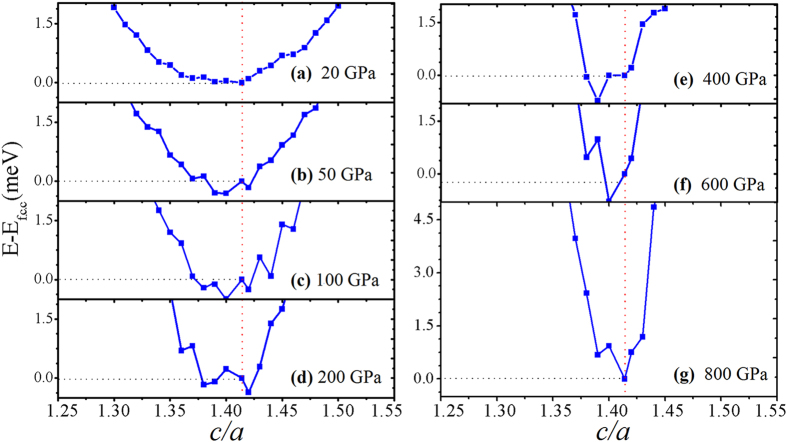
Relative energy respecting the f.c.c. phase (*c/a* = 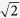) versus varying *c/a* of the b.c.t. phase of Tl at given volumes corresponding to (**a**) 20 GPa, (**b**) 50 GPa, (**c**) 100 GPa, (**d**) 200 GPa, (**e**) 400 GPa, (**f**) 600 GPa and (**g**) 800 GPa.

We have done further calculations to confirm the existence of the b.c.t phase. at high pressure reported by Kotmool *et al*. Firstly, in order to search the other possible distorted f.c.c. phases, primarily a body-centered orthorhombic (b.c.o.) phase, which has been found with more structural distortion than the b.c.t. phase, is considered. The calculations while varying *b/a* and *c/a* in range of 1.0 to 1.8 at 100 GPa and 200 GPa are reported as depicted in Fig. 3. The both contours have a diagonal symmetry. The lowest enthalpy zone is represented by the black area of contours as lay on the middle of the axes. The shape of the black zones along both the axes confirm the probability of existence of the b.c.t. phase, rather than the lower symmetry b.c.o. phase. Secondly, we also predict the high-pressure phases of Tl using USPEX code from 300 GPa to 1 TPa. At 300–600 GPa, the b.c.t. phase still prevails as the most stable phase with lowest enthalpy comparing to the f.c.c., b.c.o. and monoclinic (i.e., C2/m) phases. But the f.c.c. phase emerges to be the most stable once again at 800 and 1000 GPa. The relationships between relative energy and *c/a* at fixed volumes are calculated using FPLO with L(s)DA and FR calculation as shown in Fig. 3. The results confirm the previous reports^1^,^2^,^3^,^4^ that the f.c.c. phase (20 GPa, Fig. 4(a)) is induced by increasing pressure to be the b.c.t. phase (Fig. 4(b–f)) with a small distortion of |(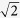
*a-c)/*c|* < 1%. Intriguingly, at 800 GPa (Fig. 4(g)), the lowest energy is corresponding to the f.c.c. phase with *c/a* = 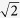 which is in agreement with the prediction. These results indicate that the perfect f.c.c. phase can be recovered by pressure of 800 GPa. This finding is quite important because it is a fulfillment suggesting that almost the III-A elements (Ga, In and Tl) exhibit the b.c.t. → f.c.c. phase transition under high pressure.

## Conclusion

In this work, we have thoroughly investigated the influence of the relativistic effects on the high-pressure phase transition of Tl. The transformation of the h.c.p. to f.c.c. phase is carried out by using various relativistic levels and exchange correlation functionals. It has been found that the relativistic effect strongly controls the transition pressure and structural parameters under high pressure. The transition pressure has been found in range of 3 to 16 GPa with the scalar and fully relativistic (SR and FR) schemes, which is in reasonable agreement with experimental result, specially with L(S)DA coupled with FR. Moreover, the SO coupling has been found to have inevitable role behind the h.c.p. → f.c.c. phase transformation. The previously reported b.c.t. phase has also been confirmed in this work. The search for high-pressure phase up to 1 TPa envisages the re-occurrence of the f.c.c. phase, that has been recovered at 800 GPa.

## Methods

We have performed all the electronic structure calculations based on density functional theory (DFT) formalism. In addition to the local spin-density approximation (L(S)DA-PW92)^16^ and generalized gradient approximation (GGA-PBE)^6^, the non-relativistic (NR), scalar relativistic (SR) and full relativistic (FR) schemes have also been considered. We have employed full-potential with local-orbital minimum-basis set implemented FPLO package^7^,^8^ and projector augmented wave (PAW)^5^ implemented Vienna *Ab initio* Simulation Package (VASP)^17^,^18^ for performing all the calculations. By using the FPLO method, the converged k-points were set to be 19 × 19 × 12, 16 × 16 × 16 and 19 × 19 × 16 for the h.c.p., f.c.c. and b.c.t. phase, respectively. For using VASP calculation, energy cutoff of 500 eV is used and the automatic k-point mesh with setting spacing between k-points of 0.2 Å^−1^ is considered (corresponding 10 × 10 × 6 and 11 × 11 × 11 for the h.c.p. and f.c.c. phase, respectively). All the setup values of VASP and FPLO methods are verified for all the calculations to ensure the energy convergence of 1 meV/atom. The third-order Birch-Murnaghan equation of state has been used to fit the energy change with volume^19^.

The structural searching method based on the evolutionary algorithm, which has been implemented in USPEX code^20^,^21^ interfacing with VASP code, has been used to extend the previous prediction up to 1 TPa (i.e., 300, 400, 600, 800 and 1000 GPa). The searches are performed by varying cell size up to 8 atoms per cell. During the prediction, number of initial randomized structures in the 1st generation is set as 40 structures. The next is carried out 30 population structures by heredity and mutations of the lowest enthalpy structure of the previous generation by 50% and 30% respectively, and remaining 20% of populations are obtained by randomization. The prediction would be done when a lowest enthalpy structure would be continuously survival within 30 generations.

## Additional Information

**How to cite this article:** Kotmool, K. *et al*. Role of relativity in high pressure phase transitions of thallium. *Sci. Rep.* **7**, 42983; doi: 10.1038/srep42983 (2017).

**Publisher's note:** Springer Nature remains neutral with regard to jurisdictional claims in published maps and institutional affiliations.

## Figures and Tables

**Table 1:** Illustration of fitting parameters consisting of volume (V_0_), bulk moduli (B_0_), and pressure derivative of bulk modulus ( 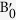 ) at zero pressure, as well as transition pressures from h.c.p. to f.c.c. phase using various calculated methods, and experiment.

Methods	P_*t*_ (GPa)	h.c.p.			f.c.c.			Reference
		V_0_(Å^3^)	B_0_(GPa)	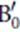	V_0_(Å^3^)	B_0_(GPa)	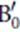	
FPLO-LSDA-NR	—	29.52	41.63	5.99	29.29	43.02	5.99	this work
FPLO-LSDA-SR	8.1	27.19	41.16	6.30	26.95	42.75	6.20	this work
FPLO-LSDA-FR	3.0	27.23	40.80	6.30	26.95	42.94	6.20	this work
FPLO-GGA-NR	—	32.62	31.59	5.60	32.27	36.22	4.84	this work
FPLO-GGA-SR	14.8	31.50	24.51	5.96	31.23	24.61	6.11	this work
FPLO-GGA-FR	8.0	31.45	25.97	5.55	31.23	24.68	6.11	this work
PAW-GGA-SR	16.8	31.96	22.21	5.91	31.39	24.63	5.84	this work
PAW-GGA-FR	8.8	29.36	38.9	4	28.84	44.78	3.94	3
ADXRD	3.5	28.7	31	4	28.2/28.8	48/50	4/3.2	3
EDXRD	4.0	28.5	35.3	6.2	28.1	29.4	6.3	1

## References

[b1] Schulte O. & Holzapfel W. B. Effect of pressure on the atomic volume of Ga and Tl up to 68 GPa. Phys. Rev. B 55, 8122–8128 (1997).

[b2] Olsen J. S., Gerward L., Steenstrup S. & Johnson E. A high-pressure study of thallium. J. Appl. Cryst. 27, 1002–1005 (1994).

[b3] Cazorla C., MacLeod S. G., Errandonea D., Munro K. A., McMahon M. I. & Popescu C. Thallium under extreme compression. J. Phys.: Condens. Matter 28, 445401 (2016). 27605357 10.1088/0953-8984/28/44/445401

[b4] Kotmool K., Li B., Chakraborty S., Bovornratanaraks T., Luo W., Mao H.-k. & Ahuja R. High pressure-induced distortion in face-centered cubic phase of thallium. Proc. Natl. Acad. Sci. USA 113, 11143 (2016). 27655891 10.1073/pnas.1612468113 PMC5056057

[b5] Blochl P. E. Projector augmented-wave method. Phys. Rev. B 50, 17953 (1994). 10.1103/physrevb.50.17953 9976227

[b6] Perdew J. P., Burke K. & Ernzerhof M. Generalized gradient approximation made simple. Phys. Rev. Lett. 77, 3865 (1996). 10062328 10.1103/PhysRevLett.77.3865

[b7] Koepernik K. & Eschrig H. Full-potential nonorthogonal local-orbital minimum-basis band-structure scheme. Phys. Rev. B 59, 1743 (1999).

[b8] Opahle I., Koepernik K. & Eschrig H. Full-potential band-structure calculation of iron pyrite. Phys. Rev. B 60, 14035 (1999).

[b9] Kenichi T., Kazuaki K. & Masao A. High-pressure bct-fcc phase transition in Ga. Phys. Rev. B 58, 2482 (1998).

[b10] Degtyareva O., McMahon M. I., Allan D. R. & Nelmes R. J. Structural complexity in gallium under high pressure: Relation to alkali elements. Phys. Rev. Lett. 93, 205502 (2004). 15600936 10.1103/PhysRevLett.93.205502

[b11] Simak S. I., Häussermann U., Ahuja R., Lidin S. & Johansson B. Gallium and indium under high pressure. Phys. Rev. Lett. 85, 142 (2000). 10991179 10.1103/PhysRevLett.85.142

[b12] Poole T. R., Kemeny P. C., Liesegang J., Jenkin J. G. & Leckey R. C. G. High resolution photoelectron studies of the d bands of some metals. J. Phys. F: Met. Phys. 3L46, L46 (1973).

[b13] Ley L., Pollak R., Kowalczyk S. & Shirley D. A. The onset of relativistic effects in the density of states of the 6s6p elements Tl, Pb, and Bi. Phys. Lett. A 41, 429–430 (1972).

[b14] Mikhaylushkin A. S., Häussermann U., Johansson B. & Simak S. I. Fluctuating lattice constants of indium under high pressure. Phys. Rev. Lett. 92, 195501 (2004). 15169413 10.1103/PhysRevLett.92.195501

[b15] Mikhaylushkin A. S., Simak S. I., Johansson B. & Häussermann U. The role of orthorhombic distortions in gallium under high hydrostatic pressure. J. Phys. Chem. Solids 67, 2132–2135 (2006).

[b16] Perdew J. P. & Wang Y. Accurate and simple analytic representation of the electron-gas correlation energy. Phys. Rev. B 45, 13244 (1992). 10.1103/physrevb.45.13244 10001404

[b17] Kresse G. & Furthmuller J. Efficient iterative schemes for ab initio total-energy calculations using a plane-wave basis set. Phys. Rev. B 54, 11169 (1996). 10.1103/physrevb.54.11169 9984901

[b18] Kresse G. & Furthmuller J. Efficiency of ab-initio total energy calculations for metals and semiconductors using a plane-wave basis set. Comput. Mater. Sci. 6, 15–50 (1996). 10.1103/physrevb.54.11169 9984901

[b19] Birch F. Finite elastic strain of cubic crystals. Phys. Rev. 71, 809–824 (1947).

[b20] Oganov A. R. & Glass C. W. Crystal structure prediction using ab initio evolutionary techniques: Principles and applications. J. Chem. Phys. 124, 244704 (2006). 16821993 10.1063/1.2210932

[b21] Glass C. W., Oganov A. R. & Hansen N. USPEX evolutionary crystal structure prediction. Comput. Phys. Commun. 175, 713–720 (2006).

